# Assessing the cost-effectiveness of waiting list reduction strategies for a breast radiology department: a real-life case study

**DOI:** 10.1186/s12913-023-09447-y

**Published:** 2023-05-23

**Authors:** Annarita Fanizzi, Elisabetta Graps, Domenica Antonia Bavaro, Marco Farella, Samantha Bove, Francesco Campobasso, Maria Colomba Comes, Cristian Cristofaro, Daniele La Forgia, Martina Milella, Serena Iacovelli, Rossella Villani, Rahel Signorile, Alessio De Bartolo, Vito Lorusso, Raffaella Massafra

**Affiliations:** 1 I.R.C.C.S. Istituto Tumori “Giovanni Paolo II”, Viale Orazio Flacco 65, Bari, 70124 Italy; 2 Direttore medico Area Valutazione e Ricerca, coordinatore del Centro regionale di Health Technology Assessment AReSS Puglia, Bari, Italy; 3 Dipartimento di Economia, Management e Diritto dell’Impresa, Università degli Studi di Bari “Aldo Moro”, Largo Abbazia Santa Scolastica, 53, Bari, 70124 Italy

**Keywords:** Cost-effectiveness analysis, Breast radiology, Quality-Adjusted Life Year, Incremental Cost-Effectiveness Ratio, Waiting list reduction strategies

## Abstract

**Background:**

A timely diagnosis is essential for improving breast cancer patients’ survival and designing targeted therapeutic plans. For this purpose, the screening timing, as well as the related waiting lists, are decisive. Nonetheless, even in economically advanced countries, breast cancer radiology centres fail in providing effective screening programs. Actually, a careful hospital governance should encourage waiting lists reduction programs, not only for improving patients care, but also for minimizing costs associated with the treatment of advanced cancers. Thus, in this work, we proposed a model to evaluate several scenarios for an optimal distribution of the resources invested in a Department of Breast Radiodiagnosis.

**Materials and methods:**

Particularly, we performed a cost-benefit analysis as a technology assessment method to estimate both costs and health effects of the screening program, to maximise both benefits related to the quality of care and resources employed by the Department of Breast Radiodiagnosis of Istituto Tumori “Giovanni Paolo II” of Bari in 2019. Specifically, we determined the Quality-Adjusted Life Year (QALY) for estimating health outcomes, in terms of usefulness of two hypothetical screening strategies with respect to the current one. While the first hypothetical strategy adds one team made up of a doctor, a technician and a nurse, along with an ultrasound and a mammograph, the second one adds two afternoon teams.

**Results:**

This study showed that the most cost-effective incremental ratio could be achieved by reducing current waiting lists from 32 to 16 months. Finally, our analysis revealed that this strategy would also allow to include more people in the screening programs (60,000 patients in 3 years).

## Introduction

Breast cancer is the most commonly diagnosed cancer in the world with an estimated 2.26 million cases registered in 2020 and is the leading cause of cancer mortality among women [1]. In recent years, breast cancer mortality has decreased both thanks to the improvement in the efficacy of therapeutic pathways and the implementation of early diagnosis strategies [2, 3]. Indeed, the benefit of screening mammography in cancer control has been established in clinical- trials and observational and modelling studies [4, 5]. The detecting of a breast cancer at an early stage allows more effective treatment and improved survival [6, 7]. Several recent study they took care of evaluating the cost-effectiveness ratio of different screening program [8–11].

The burden of the breast cancer is huge both for the National Health Service and for patients in terms of direct and indirect costs and the most cost-effective approach to manage the pathology is the mammography screening [12]. The periodicity of the screening is important and strategic to reduce the mortality and costs associated with the treatment of advanced cancers; for this reason, the timing of the screening and the related waiting lists are decisive [13].

Nevertheless, even in developed and economically advanced countries the waiting lists of a breast cancer radiology center do not allow for an effective screening program. The corporatization process of the national health system considers the rationalization of resources and clinical effectiveness in order to design a correct investment plan, that is clinically effective and economically sustainable [14, 15]. In fact, there are many economic assessments based on earned years of life and quality of reproducible life [16]. Moreover, the Italian National Health System considers both clinical effectiveness and economically sustainability unavoidable principles to ensure essential levels for health services delivery [17].

In this paper, we propose a model to evaluate different scenarios that may include optimising or increasing resources. The main goal of our work is to propose a model to evaluate the best allocation of resources that are invested in the department of breast Radiodiagnosis of Istituto Tumori “Giovanni Paolo II” in Bari, in particular, in the field of screening, in order to maximise the benefits related to the quality of care and resources employed. In particular, we intended to analyze the organization of the department of breast radiology and its costs, in order to evaluate its impact on reducing waiting lists by optimizing the resources. This is a first attempt to perform an Health Technology Assessment (HTA) approach from a public hospital-based point of view, considering the organization as a technology itself. HTA is a multidisciplinary process that determines the value of a health technology, with the purpose of informing decision-making to promote a health system that is efficient, high-quality and equitable [12]. It explores the impact of a health technology in different value domains such as clinical effectiveness, safety, costs and economic implications, organisational and environmental aspects, etc. An intervention developed to organize healthcare delivery can be considered a health technology. Indeed, in some recent works this approach has been used with reference to the reduction of waiting lists for various treatments for breast cancer [18, 19].

Regarding the first level medical examinations (visits, mammograms and ultrasounds), the current organization of the department of breast Radiodiagnosis of Istituto Tumori “Giovanni Paolo II” in Bari presents distinct waiting times based on risk factors. In this paper we propose a model aimed at improving the performances of the department in the screening tests delivery, in order to reduce waiting lists for non-cancer patients and ensuring life years gaining. We used cost-utility analysis in order to estimate the costs and health effects of the screening program, comparing two different organizational models against the current one; to this end, we used the Quality-Adjusted Life Year (QALY) for the estimation of the health effects, in terms of usefulness, of the screening strategies [20].

## Materials and methods

### Experimental data

In this study, we included the oncology patients with breast cancer registered in 2019 at the Istituto Tumori “Giovanni Paolo II” in Bari (Italy). Our database consisted of 242 patients, grouped by metastatic and non-metastatic patients.

We analyzed data according to the classification of malignant tumor internationally accepted for cancer staging, i.e. TNM Classification, so taking into account tumor size (T, measured in mm), lymph node invasion (from N0 to N3) and presence of distant metastasis (M0 and M1) [21]. Moreover, we have collected histological subtypes (ductal, lobular, tubular and mucinous) and histological grade (G, Elston–Ellis scale: 1, 2, 3).

Table 1 summarized the characteristic of the sample.

**Table 1:** Characteristic of the sample

	Absolute frequency	%
**T**		
T1A	20	8%
T1B	65	25%
T1C	95	39%
T2	59	24%
T3	3	1%
**N**		
N0	154	64%
N+	88	36%
**Histological subtypes**		
DUCTAL	189	78%
LOBULAR	44	18%
TUBULAR	5	2%
MUCINOUS	2	1%
**Histological grade**		
1	79	33%
2	109	45%
3	47	19%

### Cost analysis

In order to facilitate the allocation of resources, it is important to analyse the costs of alternative methods of providing health services [22].

The cost analysis is obtained by calculating the costs from the point of view of the National Health Service (NHS), that is, considering the direct material costs incurred by the NHS to implement the production capacity of the breast department from which we will have the reduction of waiting lists.

The costs useful to the cost-utility analysis are those related to the implementation of the screening program, which will be extended to a wider audience in the same period.

In order to carry out the cost-analysis we have considered the costs of staff, goods and services, general expenses and machinery. Our staff is composed by of 4 nurses, 4 health technicians, 3 doctors and a specialized technician.

We had the total annual cost of breast radiology staff considering the burdens and expertise of doctors, nurses, health technicians and specialized technical operators.

We have obtained the average hourly and daily costs of the individual staff, considering that a day 5 working hours are devoted to the first level exams. We have added the costs of goods and services, including diagnostic materials, surgical devices and medical materials and those related to overheads due to energy, electricity and gas. Also, of these we have calculated the average hourly and daily costs, considering the 5 working hours. Finally, we considered the costs related to the machinery, such as ultrasound, mammographs and reporting stations, also relating them to the 5 working hours.

With regard to the analysis of revenues, we have made a distinction between paying patients and patients whose services are reimbursed by the region. In particular, it is expected that 35% of the patients taking the first level examinations will pay, while the remaining ones are reimbursed by the regional health system.

### Strategies scenarios

**Fig. 1:**
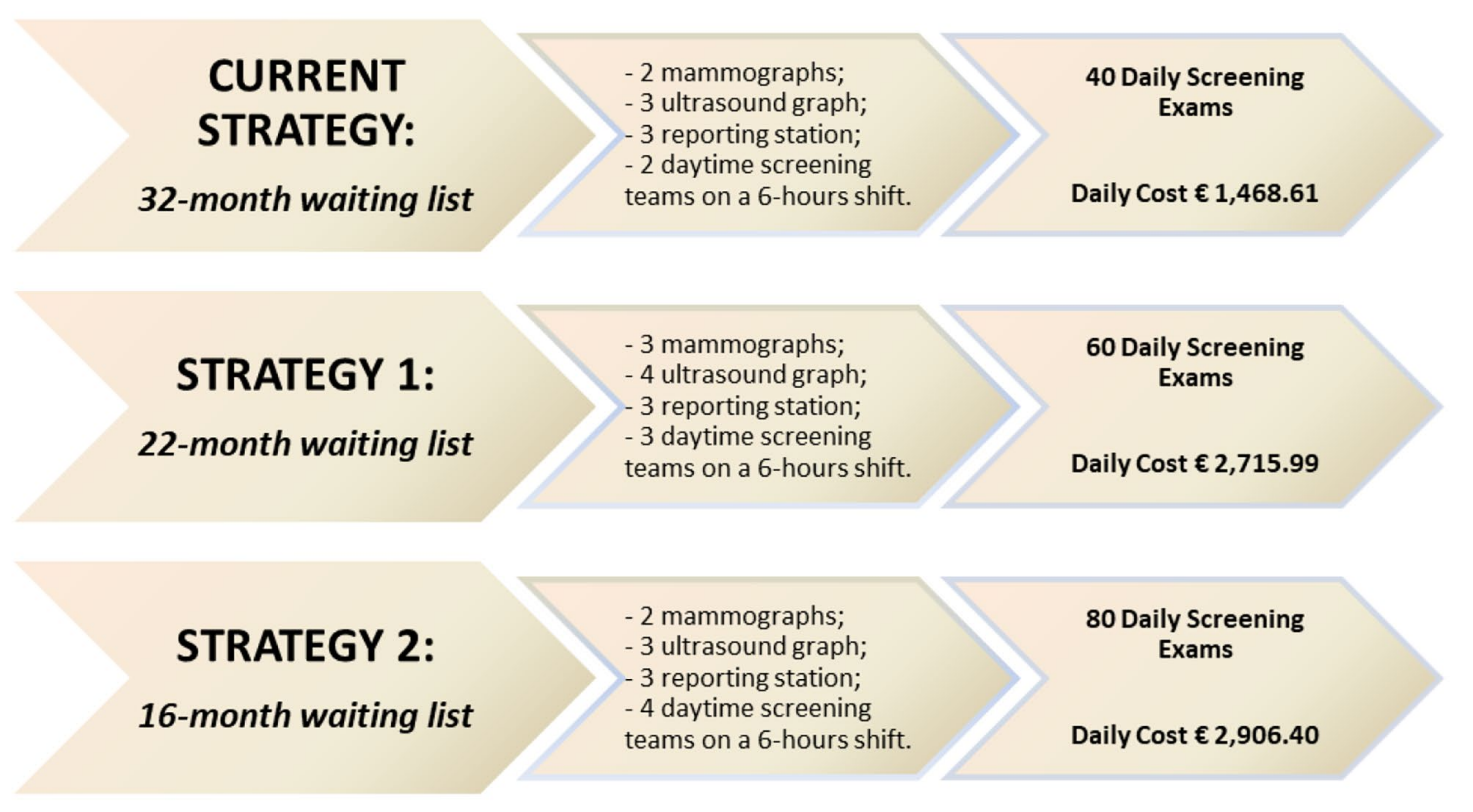
Current, first and second strategies

We have considered three scenarios related to different breast screening organizational models that we call strategies. In particular, we have compared the two new hypothetical organizational set up against the current one (Fig. 1). A health care team consisting of a doctor, a technician and a nurse can carry out 20 daily screening exams.

The current scenario is characterized by a 32-month waiting list for spontaneous screening in asymptomatic women. The resources used are:

- 2 mammographs;
- 3 ultrasound graphs;
- 3 reporting stations;
- 2 daytime screening teams on a 6-hour shift.

It produces 40 daily screening examinations with a 32 months waiting list.

The first alternative hypothetical strategy (Strategy 1) entails additional resources as:

- 1 team made up of a doctor, a technician and a nurse;
- 1 ultrasound graph and.
- 1 mammograph.

This new setting increases the output capacity of the breast department from 40 to 60 daily screening exams, with the consequent reduction of waiting lists to 22 months.

The second hypothetical strategy (Strategy 2) envisages two additional afternoon teams. This allows to increase the productivity of breast radio-diagnostics to 80 screening exams per day, halving the current waiting lists from 32 to 16 months.

### Probability of metastasizing: relationship between probability and size of tumor

The treatment of a metastatic tumor differs significantly from non-metastatic tumor in both surgery and chemo-therapy, with large differences in treatment costs and strong differences in survival, for these reasons we analyzed the probability of metastasizing.

The probability of metastasis can be calculated in terms of cells/day [23], that is, the cells that a tumor can form in 24h.

Considering 1/P as the probability of metastasis, where P represents the average number of cells that needs to form metastasis, it is possible to find a relationship between the incidence of distant metastases and the size of the tumor that is described in term of accumulated cells.

Using a Poisson distribution, it is possible to calculate a relationship between the probability of metastases and the number of accumulated cells [23].

The Poisson distribution has mean equal to µ = N(t)/P, where N(t) is the number of tumor cells accumulated at time t and calculated from the diameter of the mammographic image. This represents the average number of metastases in the population. The probability of having 0 events (i.e., no metastases) in the t-time interval is:

$$P\left(X=0\right)={e}^{-\frac{N\left(t\right)}{P}}$$.

Then the relationship between the probability of metastasizing and the size of the tumor is:

$$pr met \left(N\left(t\right)\right)=1-{e}^{-\frac{N\left(t\right)}{P}}$$

We can, therefore, estimate the probability of metastasizing and the number of metastatic and non-metastatic patients in the various T-stages of the TNM system.

We considered two thresholds – palpability and detection thresholds – for screening to be useful.

The *threshold of palpability (Tp)* indicates an average size of the tumor that patient can detect autonomously. As a result, screening, as a means of prevention, loses utility because the tumor is manifested and it may have already metastasized. Although mammography is a particularly precise instrument, it has a limit, that is, it can’t detect tumors below a certain threshold, which we call the *detection threshold (Td)*.

The study “*Computer Simulation Method for Estimating Optimal Intervals for Screening. Radiology*” [23] suggests a palpability threshold with a diameter between 25 and 30 mm, with a number of cells of approximately 10^10^ and a detection threshold of approximately 3 mm of diameter with 10^7^ cells.

### Effectiveness analysis: QALY

Patients’ life can benefit from reducing waiting lists and this benefit can be measured in terms of gained life years. To evaluate the overall effect of a medical treatment, an outcome measure that combines survival and quality of life is necessary. Quality adjusted life-years (QALY), where the quantity of life-years gained are multiplied by a weight reflecting the quality of that life, is a global measure of health which can be used for this purpose [24] The Quality-Adjusted Life Year (QALY) represents a summary measure of health outcomes for an economic assessment that considers the impact on both quantity and quality of life [20, 25]. QALY weights are constructed by valuing the health related quality of life (HRQoL) on a scale from 0 (dead) to 1 (full health) using preference-based measures.QALY is given by.

$${\rm{QALY }} = {\rm{ LYG }} \times {\rm{ QoL}}$$

where LYG is the Gained Years of Life that, in our model, are obtained by the difference between Years of Life (LYs) in one strategy and Years of Life (LYs) in the comparative strategy; QoL is a tool for expressing a quantitative assessment of the quality of life of a patient undergoing treatment or a health programme. QoL represents the weighting coefficient, which is obtained from the study on the description of HRQoL (Health Related Quality of Life) in the different stages of breast cancer [24]. This utility value is necessary to obtain a weighted estimate of the LYG, that is the years of life gained by the new screening strategies compared to the current strategy.

### Cost-effectiveness analysis: ICER

Our goal was to determine which of the strategies is the most cost-effective both in terms of qualitative survivor and cost.

The Incremental Cost-Effectiveness Ratio (ICER), a statistic used in the methodology of the Cost-Effectiveness Analysis (CEA), indicates the additional cost which derives from a unit of outcome gained by one strategy compared to another. An application of the ICER is in the Cost-Utility Analysis (CUA), in which case the ICER expresses the cost per quality-adjusted life year (QALY) gained.

In our case study, the ICER is given by the ratio of the difference in costs and the difference in effectiveness between the two new strategies and the stablished strategy [26].

The ICER expresses the cost incurred for each QALY earned, in fact it is equal to:

$${\rm{ICE}}{{\rm{R}}^1} = {{\rm{C}}^{{\rm{S1}}}} - {{\rm{C}}^{{\rm{Sc}}}}/{\rm{QALY}}{{\rm{s}}^{{\rm{S1}}}} - {\rm{QALY}}{{\rm{s}}^{{\rm{Sc}}}};$$

$${\rm{ICE}}{{\rm{R}}^2} = {\rm{ }}{{\rm{C}}^{{\rm{S2}}}} - {{\rm{C}}^{{\rm{Sc}}}}/{\rm{QALY}}{{\rm{s}}^{{\rm{S2}}}} - {\rm{QALY}}{{\rm{s}}^{{\rm{Sc}}}}$$

where.

- C^S1^ is the cost of the Strategy 1; C^S2^ is the cost of the Strategy 2 and C^Sc^ is the cost of the current strategy QALYs^S1^ are the Life Years gained and weighted for quality related to the Strategy 1 QALYs^S2^ are the Life Years gained and weighted for quality related to the Strategy 2, QALYs^Sc^ are the Life Years gained and weighted for quality related to the Current Strategy.

It is sufficient to compare the ICER of the two hypothetical strategies with the currently implemented strategy to establish the most convenient.

## Results

### Cost analysis

The administration of the Istituto Tumori “Giovanni Paolo II” provided the costs and revenues for the year 2019 (Table 2).

**Table 2:** Costs of staff, goods and services, overheads and equipment of breast radiology

	ANNUAL COSTS	Average daily cost (5 H)
Total Staff	€ 856,825.94	€ 608.28 *
Nurses and health technicians (	€ 336,429.37	
Specialized technical operator	€ 37,307.50	
Doctors	€ 483,089.07	
Goods and services	€ 225,288.23	€ 128.59
Overheads	€ 162,317.13	€ 92.65
Equipment	€ 54,000.00	

*average daily cost referred to a team member for each sanitary)

The total annual cost of the staff is € 856,825.94, from which we have obtained that on average each sanitary has a daily cost of € 608.28, considering the 5 working hours.

Goods and services have a total annual cost of € 225,288.23 and an average daily cost (5 H) of € 128.59. Overheads have an average daily cost (5 H) of € 92.65, since the total annual cost is € 162,317.13. The machines have an annual cost of € 54,000.00 and an average daily cost (5 h) of € 30.82. With regard to revenues, we have added up the revenues from the first-level examinations carried out by non-paying patients, whose benefits are reimbursed by the regional health service, and the revenues from the examinations carried out by paying patients. For paying patients, we considered the average cost of € 87.00 for first level exams, while for non-paying patients we put the cost of the ticket equal to € 36.15. Considering the characteristics of every strategy, we have obtained the costs and the revenues of the current strategy and the two hypothetical ones, as we have represented in Table 3.

**Table 3:** Revenues, costs and daily margins per strategy

	Current strategy	22-month strategy (60 patients)	16-month strategy (80 patients)
DAILY HOSPITAL’S REVENUES	€ 3,986.10	€ 5,979.15	€ 7,936.05
DAILY COST	€ 1,468.61	€ 2,715.99	€ 2,906.40
DAILY MARGIN	€ 2,517.49	€ 3,263.16	€ 5,029.65

The screening costs projected over 3 years are shown in Table 3. The administration provided unit screening costs for each strategy. In particular, the current strategy registered costs of screening equal to € 34.42 per patient, while the costs of screening of the 22-months strategy were equal to € 45.27 per patient and those related to the 16-months strategy are € 36.33.

As regards the total costs over the three years, these amounted to €1,069,687.68 for the current strategy, € 1,978,238.64 for the 22-month strategy and € 2,116,927.08 for the 16-month strategy.

**Table 4:** Screening costs per patient and total over 3 years

	*Unit screening costs*	*3-years screening costs*
32 – months (current strategy)	34.42 €	€ 1,069,687.68
22-months strategy 1	45.27 €	€ 1,978,238.64
16-months strategy 2	36.33 €	€ 2,116,927.08

By subtracting from the screening costs of each strategy, the screening costs of the current strategy we have obtained the variation of costs in each strategy.

The cost variation in the 22-month strategy is the difference between the cost of the 22-month strategy and that of the current strategy. It is equal to € 908,550.96. The change in costs in the 16-month strategy is the difference between the costs of the 16-month strategy and those of the current strategy. It is equal to € 1,047,239.40.

### Probability of metastasizing according to the T-levels and strategies

Table 5 shows the probability of metastasis related to the various T-levels in the three different strategies. For patients with a tumor classified as T1A, the probability of metastasizing is 0% in all cases.

For patients with a tumor classified as T1B, the probability of metastasizing is 3% in the 32-month strategy, 1% in the 22-month strategy and 0% in the 16-month strategy. Patients with a tumor classified as T1C have probability of metastasizing of 20% in the case of 32-month strategy, 7% in the case of 22-month strategy and 1% in the case of 16-month strategy. By introducing the palpability threshold, the probability until this threshold is 59% in case of 32-month strategy, 30% in case of 22-month strategy and 6% in case of 6-month strategy.

From Table 4 we found the number of metastatic patients at various T levels. Particularly, there are 80 metastatic patients in current strategy, i.e., 32month strategy, 62 metastatic patients in 22-month strategy and 35 metastatic patients in 16-month strategy.

**Table 5:** Probability of metastasis related to the various T-levels in the three different strategies

TNM	T1A (%)	T1B (%)	T1C (%)	T2(Inf CI) - *Palpability Threshold* (%)	*Palpability Threshold*- T2(Sup CI) (%)	T3 (%)
32month strategy Pr(CI95%)	0(0–0)	3(2–3)	20(18–21)	59(55–62)	Over *Palpability Threshold*, screening loses utility as an early diagnosis tool	
22month strategy Pr(CI95%)	0(0–0)	1(1–1)	7(6–7)	30(28–32)		
16month strategy Pr(CI95%)	0(0–0)	0(0–0)	1(1–1)	6(5–6)		

### Cost-effectiveness analysis

In order to measure the benefit that comes from reducing waiting lists, we calculated years of life and Years of Life Gained by means of the method above described. The availability of data allowed us to set the analysis in a medium-term time span of 3 years from diagnosis. The current strategy includes 30,000 patients in 3 years, while the 22-month and 16-month strategy respectively provide 45,000 and 60,000 patients in 3 years (Table 6).

**Table 6:** Total number of patients screened over 3 years: Incident patients distributed per metastatic/non-metastatic condition and screening strategy

		Incident patients	Patients in 3 years
32 months - Current strategy	*metastatic*	12.8 (12.5–13.2)	30,000
Pr(CI 95%)	*non-metastatic*	25.9 (25.7–26.1)	
22-months strategy	*metastatic*	14.9 (14.7–15.1)	45,000
Pr(CI 95%)	*non-metastatic*	43.2 (43.1–43.3)	
16-months strategy	*metastatic*	11.2 (11.1–11.3)	60,000
Pr(CI 95%)	*non-metastatic*	66.3 (66.2–66.4)	

Multiplying incident patients by life expectancy, obtained from the Surveillance, Epidemiology, and End Results (SEER) survival by disease stage database [27], we found Life years (LY) for metastatic and non-metastatic patients and for all patients in every screening strategy. Comparing the LYs of each strategy with those of the current strategy we obtained the Years of Life Gained (LYGained). In 22-months strategy the LYG is equal to 47.8. We obtained it by the subtraction between LYs of 22-months strategy and LYs of current strategy. In 16-months strategy, instead, the LYG is equal to 103.8. We had it by the subtraction between LYs of 16-months strategy and LYs of current strategy. The 32-months current strategy returned QALY of 60.9, the 22-months strategy reported a QALY of 96.4, whereas the 16-months strategy returned QALY of 138.4.

The study “Health related quality of life in different states of breast cancer, Qual Life Research” [11] suggests Quality of Life (QoL) indices of 0.696 in the first year of disease and 0.779 in subsequent years in the non-metastatic stage. While the QoL index for metastatic disease is 0.685 in all years of the disease. Multiplying the LYG by their quality, through QoL indices, we got QALY for each strategy by differencing for metastatic and non-metastatic stage (Table 7).

**Table 7:** QALY for each screening strategy, highlighting differences by metastatic and non-metastatic stage

		QALY
32 months current strategy	*metastatic*	10.3 (10.0-10.7)
	*non-metastatic*	50.6 (50.2–51.0)
22-months strategy	*metastatic*	12.0 (11.7–12.3)
	*non-metastatic*	84.4 (84.4–84.5)
16-months strategy	*metastatic*	9.0 (8.8–9.2)
	*non-metastatic*	129.4 (129.1-129.8)

The current strategy obtained a QALY of 10.3 in the metastatic case, while 50.6 in the non-metastatic case. In the 22-months strategy, QALY in the metastatic case is equal to 12.0, while in the non-metastatic case it is equal to 84.4. The 16-months strategy obtained a QALY of 9.0 in the metastatic case, while 129.4 in the non-metastatic case. By comparing the QALY of each strategy with the current strategy we obtained the incremental QALY (QALYs).

The 22-months strategy returned QALYs of 35.5, subtracting the QALY value of the 22-months strategy from that of the current strategy. The 16-months strategy, however, reported a QALY of 77.5, obtained from the difference between the value of the QALY of the 16-months strategy and that of the current strategy.

The Incremental Cost-Effectiveness Ratio (ICER) is given by the ratio between the variation of costs and the difference in effectiveness between the two new strategies each of which compared against the current one (Table 8). In particular, we obtained the ICER’s 22-months strategy equal to €25.614,30 and the ICER’s 16-months strategy equal to €13.507,31.

**Table 8:** Incremental Cost-Effectiveness Ratio (ICER) for each screening strategy

	*3-years screening costs*	QALY	ICER (Δ€/ ΔQUALY)
32 months current strategy	€ 1,069,687.68	60.9	-
22-months strategy	€ 1,978,238.64	96.4	25.614,30
16-months strategy	€ 2,116,927.08	138.4	13.507,31

## Discussion

Screening is the only effective tool to prevent breast cancer. The timing of screening and the related waiting lists are crucial. Reducing waiting lists can decrease the costs of the health system and improve the quality of life [28]. A prudent governance of the hospital should implement programs of reduction waiting lists, not only to pursue the social object of people’s care, but also to pursue purely corporate purposes of minimizing costs; for this reason we want to propose a model that can evaluate possible strategies in order to optimize and increase resources. A hospital based HTA approach can be implemented by evaluating organizational models as technologies and to especially investigate the use of economic and human resources in order to define optimal strategies to purse clinical effectiveness and sustainability [18].

Some studies have shown that considering the cost of the program and the gained QALYs, the performance decreases when screening is offered to women with lower risk [8, 10, 11];. Risk-based strategies can reduce harm and costs, therefore accurate measures of individual risk need to be developed to implemented opportunely strategy [29].A population approach and risk stratification, therefore, can be useful. The analysis of the data began with the search for a method of calculating the reduction in waiting lists for mammographic screening in accordance with the effects in terms of cost-effectiveness. The results we have obtained were raw data for cost-benefit analysis. It was therefore necessary to reformulate the results in terms of QALY and to allocate the relative cost per strategy. To obtain the gains in QALY from different strategies, we acted with more intense screening programs, but treating patients, metastatic and not, with the necessary care. The number of metastatic patients, despite the reduction of waiting lists from 32 to 22 months, does not decrease but increases. This is caused by the increased participation of patients in the breast screening programme, which leads to an increase in incidents.

The most expensive strategy is the 16-month waiting list strategy. Nevertheless, it could be the most cost-effective considering the high reduction in the incidence of metastatic patients.

The cost-utility analysis allows to compare different interventions using a homogenous unit of measure: the QALY. From the comparison of the ICER – Incremental Cost-Effectiveness Ratio, the final indicator of the cost-benefit analysis – of the two hypothetical strategies with the currently implemented strategy, we observed that the 16-months strategy is dominant, while the 22-months strategy is less cost-effective, perhaps due to cost inefficiencies. If we hadn’t had a comparator, both strategies would have been below the cost threshold for QALY indicated in the national guidelines [4]. In this paper we have proposed a cost-effectiveness analysis workflow aimed at identifying a waiting list reduction strategy, with strict reference to the company situation. Following the proposed analysis scheme, the optimal scenario resulted to be the immediately more expensive one, but which over the course of three years proved to be more convenient and effective. Clearly, the result achieved is linked to the economic-managerial situation of the ‘John Paul II’ Cancer Institute, but if the analysis carried out is suitably adapted to the individual situations of the healthcare institutes, it can represent a highly useful management tool both for the reduction of the costs of the national health service and for the improvement of the quality of life of the patients.

## Conclusions

Breast cancer stages influence treatment of patients; this means that earlier detections could imply better chances of survival and a greater probability to have success after treatment [30].

Waiting lists at mammographic screening should have a periodicity lower than the interval between the palpability threshold and the detection threshold (about 4.6 years) to create utility. However, not all reductions would lead to cost-effective improvement. In particular, implementing inefficient strategies on the organizational side would involve unnecessary expenditure of resources and, therefore, a negative evaluation of the strategy.

A first approach to hospital based HTA has been implemented in the breast radiology department of Ospedale Oncologico Giovanni Paolo II di Bari, by evaluating organizational models as technologies and to especially investigate the use of economic and human resources in order to define optimal strategies to purse clinical effectiveness and sustainability.

The study leads to the conclusion that the strategy of reducing waiting lists to 16 months is the most cost-effective and so, it can be considered dominant. Reducing current waiting lists from 32 to 16 months requires an investment of resources in the healthcare system of € 1,047,239.40 particularly addressed on a better use of Professionals, more than on additional Medical Devices.

This strategy, in addition to contributing to greater clinical-corporate effectiveness of the department, allows to include more people in the screening programs (60,000 patients in 3 years).

On the other side, this organizational solution will need to be flanked by increasing activities of promoting breast cancer prevention, to avoid that the department effort to improve its capacity in screening test delivery could fail his healthcare outcome.

## Data Availability

The datasets used and/or analysed during the current study available from the corresponding author on reasonable request.
